# A qualitative study of lived experience perspectives and experiences of eating disorder treatment with ANZAED Credentialed Eating Disorder Clinicians

**DOI:** 10.1186/s40337-026-01529-6

**Published:** 2026-02-03

**Authors:** Felicity Martin, Janet Conti, Madalyn McCormack, Gabriella Heruc, Katarina Prnjak, Rebecca Barns, Phillipa Hay

**Affiliations:** 1 School of Psychology, Western Sydney University, Penrith, Australia; 2 Translational Health Research Institute, School of Medicine, Western Sydney University, Locked Bag 1797, Penrith, 2751 Australia; 3 Eating Disorders and Nutrition Research Group, Translational Health Research Institute, School of Medicine, Western Sydney University, Penrith, Australia; 4 Graduate School of Health, University of Technology Sydney, Sydney, Australia; 5 Mental Health Services, Campbelltown Hospital, Campbelltown, Australia

**Keywords:** Eating disorders, Credential, Lived experience, Continuing professional development, Treatment preferences, Qualitative

## Abstract

**Background:**

The ANZAED (Australia and New Zealand Academy for Eating Disorders) Eating Disorder Credential is the first national, cross-disciplinary program to recognise minimum standards qualifications, knowledge, training, and ongoing professional development for health professionals to provide safe and effective treatment. While timely access to safe and effective treatment is known to improve quality of life and increase the likelihood of optimal treatment outcomes, there is currently no empirical evidence on the Credential’s impact for people with an eating disorder (ED). To address this gap, this study explored the perceptions and treatment experiences of individuals with an ED who received care from a Credentialed Eating Disorder Clinician.

**Methods:**

Participants were 16 people with lived experience of an ED, who had received treatment from a credentialed clinician. Participants engaged in a semi-structured interview and an online self-report survey, both exploring their ED treatment experiences. Analysis included descriptive statistics from survey data and an inductive reflexive thematic analysis of interview transcripts.

**Results:**

The first theme generated by the thematic analysis was treatment experiences with credentialed vs. non-credentialed clinicians, with (1) trust and safety, (2) seeing the whole person, and (3) teamwork identified as subthemes. The second theme was attitudes towards the Credential, with (1) the perception of the Credential as a source of hope and (2) the potential for improved access to appropriate treatment as subthemes.

**Conclusions:**

Participants consistently perceived treatment with credentialed clinicians positively and felt that additional training and supervision facilitated trust in credentialed clinicians. Some participants reported their treatment team consisted of credentialed clinicians working together to coordinate treatment, which was also perceived to facilitate trust. However, some participants felt uncertain about whether the Credential met their needs and instead emphasised the importance of treatment access in regional locations and an understanding of individual presentations including comorbid conditions.

**Supplementary Information:**

The online version contains supplementary material available at 10.1186/s40337-026-01529-6.

## Background

In recent years, there have been reforms to the availability of, and access to, evidence-based treatment for eating disorders (EDs) in Australia. Due to their typically complex course [1], EDs may become chronic and disabling in nature and can have a profound impact on physical and mental health [2, 3]. One initiative that aims to improve access to and ensure the quality and safety of ED treatment is the Australia & New Zealand Academy for Eating Disorders (ANZAED) Eating Disorder Credential [4, 5]. The Credential is a multidisciplinary credential open to dietitians, general practitioners, and mental health clinicians in Australia who have the qualifications, knowledge and ongoing professional development needed to meet a set of minimum standards considered necessary for the delivery of safe and effective ED treatment [4–6]. To become credentialed, clinicians need to have at least two years of clinical practice and to complete an NEDC approved course of introductory and treatment provision training. Some early applicants were able to provide written evidence of their prior experience and training instead of completing the specified training under a “sunset clause” which was available from November 2021 and June 2022. To maintain the Credential, clinicians are required to complete a minimum of 15 h of ED specific continuing professional development (CPD) per year, including 6 h of ED specific supervision. The Credential appears to be the first ED specific credential nationally. While the International Association of Eating Disorders Professionals (IAEDP) offers a certification program for ED professionals, it has yet to gain traction in Australia, has not received consistent endorsement from other international professional organizations, and its effectiveness has not been evaluated empirically [7].

For individuals with EDs, prompt access to effective, evidence-based treatment can lead to full recovery and/or improve their quality of life [8].There is evidence that a multidisciplinary approach to treatment, including psychological, medical, and dietetic involvement is needed to optimise chances of recovery [9] and therefore a multidisciplinary approach is recommended by practice guidelines and standards across Australia [10], the United Kingdom [11], and the United States [1]. As the Credential is open to multiple disciplines, it may improve the nature of multidisciplinary care in Australia by encouraging common standards and goals between professionals.

As part of the development of ANZAED’s credentialing system, in-depth consultation with stakeholders was sought. This consultation found that both people with lived experience and professionals perceived that the introduction of a credentialing system would be beneficial overall, with benefits including assuring quality treatment, increasing the chance of a positive first contact for people seeking treatment, improving access to care, and keeping treatment recipients safer [12]. Participants with lived experience of EDs viewed the ability to choose professionals themselves through a Credential website as empowering. They emphasised the need to be able to search by practical characteristics such as Medicare status, therapist demographic characteristics such as gender and language spoken, and clinical specialisation. Participants with lived experience also valued access to resources within a Credential website that included explanation of access to different treatment options [12].

Consistent with evidence-based practice [13, 14], the integration of client preferences into the rollout of the ANZAED credentialing system is anticipated to improve treatment access, uptake and outcomes. This would also align with Peterson et al.’s [15] suggestion that further understanding and integration of client preferences into evidence-based practice may improve ED treatment outcomes. The connect·ed website (https://connected.anzaed.org.au/) for the Credential has integrated feedback of people with lived experience of EDs through inclusion of a search function that allows users to search for credentialed clinicians, with filters including location, gender, areas of interest, waiting list status, profession, languages spoken, practice sector and practice provider options, including in person or Telehealth services [4]. These filters may improve access to suitable ED treatment interventions that are personalised to client needs, while also empowering the person to choose professionals that align with their preferences [16]. The website also contains resources to help treatment seekers navigate and understand treatment pathways.

While the development and implementation of the Credential builds upon previous research into expert recommendations and the needs of clinicians and consumers, there is limited empirical evidence examining the benefits of credentialing professionals both in the ED field and non-ED related fields, despite general consensus on their value. There is also little empirical evidence assessing how credentialing impacts the individuals with lived experience of EDs who receive care from credentialed clinicians. As such, the impact of the ANZAED credentialing system on outcomes for those with EDs remains unclear.

In order to verify the efficacy of the Credential in ensuring minimum standards are met and improving access to ED treatment, it is important to understand the experiences of those living with an ED who have been treated by credentialed clinicians. The importance of including lived experience in research and program development is increasingly acknowledged as having the potential to drive more meaningful and effective outcomes. However, in the field of eating disorders, co-designed research involving consumers, clinicians, and researchers across the entire research journey is scarce [15, 17, 18]. This may be due to several potential challenges when working with vulnerable populations including ethical concerns around the risk of harm or relapse [18]. Despite these concerns, emerging evidence suggests that ethical engagement with individuals who have lived experience is both feasible and valuable. An example of this is the development of a consumer checklist to support individuals to locate evidence-based treatment that was co-designed by researchers and people with lived experience of an ED and later evaluated by people with lived experience and clinicians [19]. The present study aims to explore the treatment experiences of people living with an ED with credentialed clinicians. This includes accessibility of care, timeliness of intervention, treatment experiences, and perceived therapeutic outcomes. Due to the novelty of the Credential and the lack of prior research regarding the implications of professional credentials, a qualitative approach was used to allow for an in-depth exploration of the experiences of individuals who have accessed ED treatment from credentialed clinicians.

## Methods

### Design

This qualitative study used an inductive reflexive thematic analysis of semi-structured interviews with people with lived experience of eating disorders who reported having received treatment from credentialed clinicians. The interviews focused on participant experiences of treatment and their perceptions of the Credential. Demographic and clinical characteristics of the sample were also pooled from the participants’ responses to closed ended survey questions.

### Participants

Participants were 16 individuals with lived experience of an ED who had completed a self-report survey about their treatment experiences and perceptions of the Credential. All participants who had indicated on the survey that they (1) were interested in participating in a semi-structured interview; and (2) had experienced treatment with a credentialed clinician were interviewed. All participants identified as female with a mean age of 32.06 years (SD = 8.35), and 12 participants (75%) reported that their cultural and ethnic background was Oceanian. All participants lived in the eastern states of Australia (New South Wales, Queensland, and Victoria) and most (81.3%) lived within a metropolitan setting. The majority of participants reported being single (68.8%) and having a tertiary education (81.3%).

### Procedure

This study was approved by the Western Sydney University Human Research Ethics Committee (approval number: H15252).

Participants were asked open-ended questions in a semi-structured interview, exploring their perception of the value of treatment from a credentialed clinician, how the credentialing system impacted their access to evidence-based care, and how their overall treatment experience compared to previous treatment experiences (See Additional File 1 for the interview questions). Interview questions invited both positive and negative experiences. The interviews lasted approximately 40–50 min. They took place as online audiovisual meetings and were recorded and transcribed using Zoom’s automatic transcription feature. These transcripts were then de-identified and edited. De-identified transcripts were emailed to participants to member-check for accuracy and to invite them to remove further information perceived to be potentially identifying. Following transcription, additional clarifying questions were emailed to some participants, to confirm any missing details, gain a deeper understanding of their intended meaning, or invite them to elaborate further. In addition to the transcripts, responses to these questions were also included in the thematic analysis.

The Hospital Anxiety and Depression Scale (HADS) [20] and Eating Disorder Examination - Questionnaire Short (EDE-QS) [21] were completed by participants as part of the initial self-report survey. The EDE-QS is a screening measure for ED symptoms over the past 7 days, where a cut off score of 15 indicates that an individual is experiencing a clinically significant level of ED symptomatology [21]. The HADS is a measure of anxiety and depression, where a cut off score of 8 on the anxiety or depression subscale indicates clinically significant symptoms [20].

### Analysis

Interview transcripts were analysed using the six phases of reflexive thematic analysis, as described by Braun and Clarke [22, 23]. Through the inductive reflexive thematic analysis, themes were developed in response to the data itself in order to emphasise meaning generated by the participant. The explicit language used by participants, as well as analysis of their implicit meaning, was used to construct themes. A constructivist approach was used, where the researcher’s understanding of the meaning of the data was constructed through their engagement with explanations of experiences given by participants and discussions with the research team to expand the richness of interpretations. This approach was chosen to utilise the subjectivity of participant responses, as their subjective experiences are relevant to the research question and aims. Additionally, the paucity of research on the effects of treatment by credentialed clinicians on patient experiences means that inductive analysis is appropriate to develop a thematic map of these effects.

For the thematic analysis, transcripts were first read to gain familiarity with their content while noting any immediate observations (FM). Transcripts were then reviewed again, while coding meaningful sections of data using NVivo. These codes were a combination of descriptive and interpretive labels, as the authors were interested in both semantic and latent data. These codes were combined into overarching themes and subthemes to develop emerging themes in discussion with authors JC and PH. These initial candidate themes were reviewed and refined by all authors to ensure that each theme was coherent, heterogeneous to other themes, and relevant to the research question. For each theme, exemplar quotes were selected and analysed as outlined in this report (see Additional File 2 for exemplar extracts). A thematic map was created to visualise the relationships between themes and their associated data items.

### Researcher positioning statements

Felicity Martin is a nonbinary, Anglo-Australian provisional psychologist who completed this research as part of a Master of Clinical Psychology. They are interested in giving a voice to lived experiences through qualitative research. Felicity Martin was not involved in the interview process, and the analysis was based on collected responses. Janet Conti is a female, Anglo-Australian clinician and researcher whose research is focused on innovating ED treatments through drawing on the wisdom of people living with an eating disorder and their support networks. Her interest in the broadening of treatment options for people experiencing EDs may have increased her responsiveness to participant quotes in this research that indicated the need for broadening the ED treatment options. Phillipa Hay is a cisgender female psychiatrist and Credentialed Eating Disorder Clinician with a long-standing clinical and research commitment to improving the understanding of the experience of an eating disorder and its treatment. She was formally trained first in psychodynamic psychotherapy and then in cognitive behaviour therapy. She has experience of caring for many people with eating disorders in general hospitals and outpatient private practice setting, has been a lead investigator on several clinical trials and lead author on Australian guidelines endorsing the need for new person centered and flexible approaches in care. Madalyn McCormack is a female, Anglo-Australian research officer, psychologist, and Credentialed Eating Disorder Clinician. Her clinical experience working with adults with eating disorders and other mental health concerns may have influenced her interpretation of participants narratives of their treatment experiences. Katarina Prnjak is a female provisional psychologist with 7 years of experience conducting research in the field of EDs. Her experience working in a multidisciplinary team may have increased responsiveness to quotes mentioning the value of teamwork. Rebecca Barns is a female, British clinical psychologist and researcher living and working in Australia. Her work focuses on trauma-informed and evidence-based approaches to eating disorders and related mental health difficulties, including integrating lived experience perspectives and body-based practices, such as yoga, into treatment and recovery frameworks. Her commitment to person-centred and embodied approaches to care may have shaped her attentiveness to participant narratives and interpretation of themes that highlighted the importance of safety, agency, relational attunement and connection within treatment, reflecting her belief in the value of holistic, flexible, and compassion-focused approaches in supporting recovery. Gabriella Heruc is a female dietitian and researcher with over 20 years working in the ED space. As the Credentialing Director for ANZAED, she was involved in the development and implementation of ANZAED and is passionate about ensuring its success. This may have increased her focus on responses about the perceived strengths of the Credential as well as areas for future improvement to support the Credential’s sustainability.

## Results

### Clinical characteristics

The clinical characteristics of participants are shown in Table 1. The majority of participants self-reported as currently experiencing ED symptoms (*n* = 12, 75%), while all participants’ scores on the EDE-QS met the cut off for clinically significant ED symptoms (*n* = 16, 100%). All participants exceeded the cut off score for the anxiety and depression subscales of the HADS, indicating clinically significant anxiety and depression (*n* = 16, 100%). Most participants (*n* = 15, 93.8%) had received a previous and/or current diagnosis of AN; 5 participants (31.3%) had received a previous and/or current diagnosis of BN; and 6 participants (37.6%) had received a current and/or previous diagnosis of another ED, including BED and OSFED. All participants reported a longstanding course of an ED, with over three years since reported diagnosis.

**Table 1:** Clinical characteristics of participants (*n* = 16)

Characteristic	Frequency	Percent
Experience of ED Symptoms		
Currently experiencing	12	75%
Diagnosed EDs^a^		
Anorexia nervosa	15	93.8%
Other diagnosis (BN, OSFED, BED)	11	68.9%
Comorbid conditions^b^	12	75%
ED treatment received from a credentialed clinician^a, c^		
Currently receiving treatment for ED	13	81.3%
Dietitian	13	81.3%
Psychologist	10	62.5%
Other	13	81.8%
EDE-QS Score		
≥ 15	16	100%
HADS Score		
Anxiety ≥ 8	16	100%
Depression ≥ 8	16	100%

^a^These are not mutually exclusive

^b^Comorbid conditions were self-reported in the interview and included personality disorders (e.g. Borderline Personality Disorder), neurodevelopmental disorders (e.g. Autism), trauma and stressor related disorders (e.g. Posttraumatic Stress Disorder), substance use disorders, injuries (e.g. knee injury) and digestive disorders

^c^Other included mental health nurse, nurse practitioner, psychotherapist, psychiatrist, social worker or GP

### Thematic analysis

**Fig. 1:**
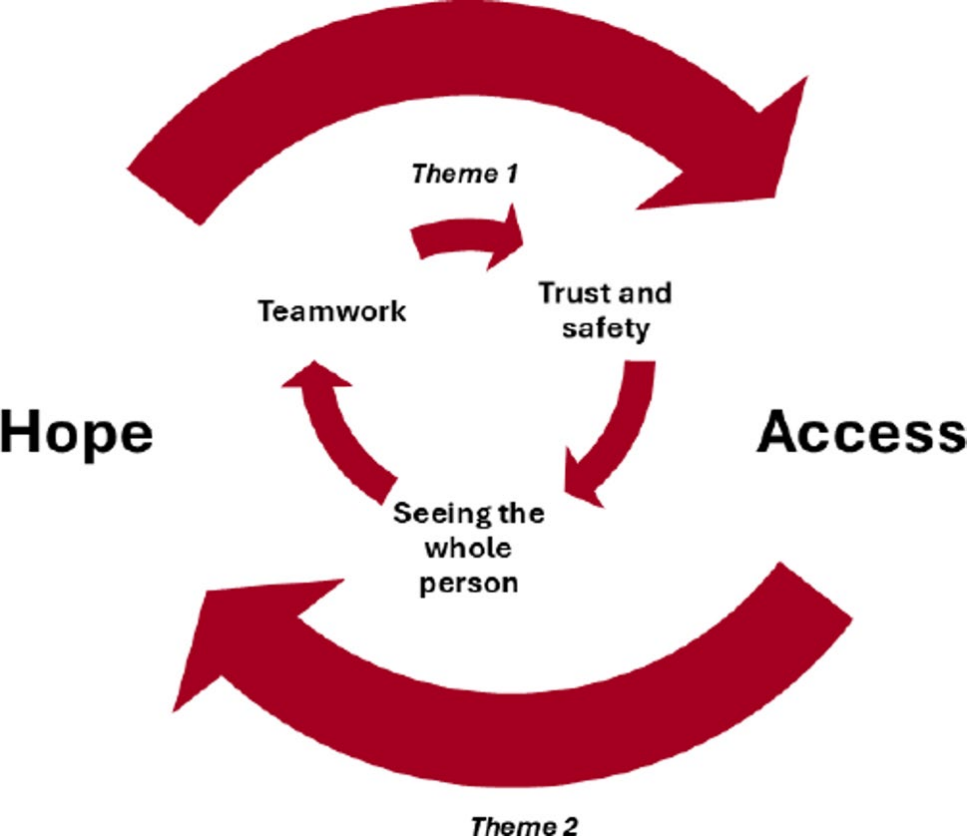
Experience and perceptions of treatment with a Credentialed Eating Disorder Clinician

Analysis of participants’ experiences and perceptions of treatment with credentialed clinicians generated two main themes with nested subthemes (Box 1) with their inter-relationship depicted in Fig. 1.

### Box 1: Experiences and perceptions of treatment by credentialed clinicians


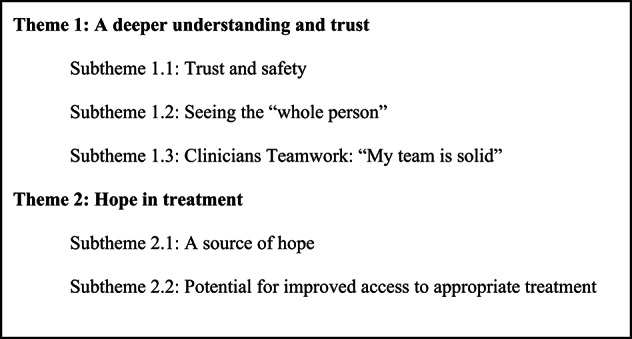


**Theme 1: A deeper understanding and trust**

Overall, participants consistently reported positive experiences of treatment with credentialed clinicians noting a deeper understanding of EDs, a sense of trust in their clinician’s commitment to learning about EDs and reduced weight bias when discussing diet and exercise. Participants reports of experiences with non-credentialed clinicians however were more varied. Differences between experiences with credentialed and non-credentialed clinicians, as described by participants, clustered around three subthemes, including trust and safety facilitated by knowledge (subtheme 1.1), seeing the “whole person” (subtheme 1.2), and clinicians’ teamwork (subtheme 1.3).

**Subtheme 1.1: Trust and safety**

Participants described treatment experiences that contributed to a sense of trust and safety including a sense of validation when treated by credentialed clinicians. This was frequently attributed to the deeper understanding of EDs held by these clinicians. For example, one participant reported “it kind of makes me feel safer and more confident knowing that I’ve got the right person […] to help me” (P1). This sense of safety was described by another participant as allowing her to “trust them to manage [her] weight” (P3). Another participant emphasised the importance that they did “not feel judged” and “validation, especially in those early stages when I didn’t feel sick” (P5). These participant experiences highlight how treatment with a credentialed clinician both validated and took seriously the ED and in doing so created a sense of trust and safety in the treatment for the participants.

In contrast, several participants reported feeling invalidated or dismissed by non-credentialed clinicians, resulting in a minimisation of the need for specialised ED treatment. For example, one participant recollected that a non-credentialed clinician minimised their experience in the suggestion “just don’t buy cake” and that “things kind of escalated from there” (P2). Other participants described experiences of a lack of concern from non-credentialed clinicians that contributed to decreasing their motivation to recover and invalidating their experience through being positioned as not “sick enough” where they “needed to make myself more unwell” (P10) to qualify for ED treatment. Many participants had described past experiences of ED symptoms not being prioritised or taken seriously, with one participant describing her struggle to find treatment as leaving her “confused and dazed and demoralised” after feeling that she was seen as “too sick, or not sick enough” (P12). Participants also noted experiences of non-credentialed clinicians who focused on their weight, while not attending to other physiological or psychological ED symptoms. Furthermore, participants in larger bodies reported being encouraged by non-credentialed clinicians to lose weight despite seeking treatment for their ED, while other participants reported concerns being dismissed due to being considered a “healthy weight” (P11). These participants noted that credentialed clinicians had different “perspectives on weight, size, shape, BMI, and the fact that bodies change” that allowed for a greater sense of safety and validation in treatment (P8). These responses indicated that these experiences with non-credentialed clinicians’ polarised between either minimising the ED symptom or an overfocus on ED symptoms/weight loss with a neglect of the psychological dimensions of the experience.

**Subtheme 1.2: Seeing the “whole person”**

Most participants talked about the importance of treatment being individualised to their needs, and of being seen as a “whole person” (P14), rather than clinicians purely following “a pathway that’s written in a manual” (P7). Participants also described appreciation for flexible approaches to treatment that recognised their unique situations.

Most participants described one or more comorbid conditions that they lived with, including other mental health and/or physical conditions. Several participants discussed the interaction between the ED experience and these comorbid conditions, with some participants describing the ways that these conditions prevented them from accessing appropriate inpatient treatment due to their needs conflicting with expectations and facilities typical of ED treatments. One participant described her credentialed dietitian actively working to problem solve through hearing her concerns with the ED and other health conditions (P1). In contrast, one of this participant’s previous, non-credentialed dietitians suggested a restrictive diet, “almost like a keto diet,” rather than addressing concerns around how restrictive eating perpetuates patterns of binge eating.

Several participants described a history of trauma, explaining that their non-credentialed clinicians “didn’t feel very comfortable working in that area” (P6) or focused on trauma instead of ED symptoms (P1). On the other hand, one participant described her credentialed psychologist being flexible and willing to revisit “if you’re having a flashback […], how to handle that” because “that will help [the client] eat” (P4). Another participant described her credentialed psychologist using a trauma informed approach that “helped make sense of a lot of things, even like the physical disability and pain pieces” (P9). These participant experiences highlight the importance of credentialed clinicians having skills in working with the complex and unique needs of the person. They also emphasise that treatment should not be limited to the ED nor excluding the ED from a comprehensive and holistic treatment approach.

**Subtheme 1.3: Clinicians Teamwork: “My Team is solid”**

Most participants reported experiencing treatment with two or more credentialed clinicians and described their clinicians working together as a team. Four participants had experienced treatment from a credentialed psychologist and dietitian, while another five participants had three or more credentialed clinicians on their treatment team. A participant whose team included a GP with a credentialed psychologist, psychiatrist and dietitian reported feeling a sense of safety due to knowing her team were working together to coordinate her care (P3). This sense of safety was echoed in the following extract:

Extract 1:

*It feels safe*,* and also incredibly frustrating*,* but in a good way. It feels reassuring to hear the same information coming from all team members. The direction is clear and some of the uncertainty […] is decreased. I feel held*,* supported and even though my internal world is in chaos*,* at least my team is solid. (P6)*

This extract highlights how safety was cultivated by consistency between team members that contributed to the sense of being “held” thereby not reproducing a parallel process of “chaos” in their internal world.

Participants described consistency in teamwork between credentialed clinicians with one describing this as being “on the same page” (P16). One participant described her credentialed dietitian and psychologist communicating with each other and with her non-credentialed GP, which she felt saved her from having to “re-explain things seven times” and prevented clinicians from contradicting each other (P15). This participant also felt that working with credentialed clinicians had helped her access “links that really help you to get the right treatment as early as possible”. However, another participant noted that while the credentialed staff at an ED treatment centre “talk to each other and they send letters to my doctors and psychiatrists”, her GP “doesn’t read them unless he has to” (P4). Another participant described her credentialed psychologist and dietitian having regular phone and email contact with her GP (P6).

Participants also described teamwork between their clinicians as encouraging honesty and trust. For example, when describing teams of non-credentialed clinicians, two participants described being able to use inconsistent communication to “play [their clinicians] off” (P3) against each other to “work the system” (P7). On the other hand, participants who described regular communication between credentialed clinicians created a safety net that prevented them from hiding ED symptoms or behaviours from their clinicians (P6), and facilitated sharing information, particularly when they were comfortable with this (P16).

**Theme 2: Hope in treatment**

In light of their positive treatment experiences with credentialed clinicians, all the participants perceived the ANZAED Eating Disorder Credential positively. Most indicated that they would recommend Credentialed Eating Disorder Clinicians and the connect·ed searchable directory to others looking for ED treatment. Many viewed the Credential as a way to improve their access to clinicians who have reached a minimum level of understanding of EDs, who could help them access safe and appropriate treatment. Several participants expressed feeling that the Credential showed a clinician’s commitment to and prioritising of the needs of individuals with EDs, giving them a sense of hope for the future. Thus, attitudes towards the Credential focused around the Credential as a source of hope (subtheme 2.1) and the potential for improved access to appropriate treatment (subtheme 2.2).

**Subtheme 2.1: A source of hope**

Several participants reported that knowing about the Credential and the requirements of credentialed clinicians cultivated a sense of hope. These participants perceived that the development of the Credential indicated that their needs have been taken seriously and contributed to a sense of hope that there are clinicians who care about supporting them. Participants spoke about the Credential indicating a sense of commitment to understanding EDs, which they linked to being able to place trust in clinicians.

Extract 2

*The benefit of a credentialing program is that it says to eating disorder patients on a higher level that there are people out there who want to work with you. That you’re interesting enough and valid enough. […] there are clinicians who have devoted a significant time to making sure they have the skills to help you. […] this credentialing system is there because [ED patients] are important and their needs are unique*,* and that these people have spent considerable time learning how to help you as an individual with an eating disorder […] these people care about you. (P12)*

Implicit in the Credential for this participant was the sense that people living with EDs are worthy of treatment that meets their needs and preferences and is delivered by clinicians who “want to work with you” and “care”. This ethic of care was a source of hope for the participants.

**Subtheme 2.2: Potential for improved access to appropriate treatment**

Participants felt positive about the idea of the Credential as a measure to ensure that clinicians are “qualified to at least know the basics around how to treat someone with an eating disorder” (P1) and that “at least you would kind of know that they were probably getting adequate treatment as opposed to guesswork” (P5). Some suggested that this could be helpful for people who are early in their help seeking by ensuring that they do not encounter “misleading advice” that might “reinforce […] unhelpful ideas and thoughts” (P10) thereby risking harm. While several participants considered early diagnosis and intervention to be important for someone experiencing an ED, participants also noted the challenging nature of finding clinicians to seek help from. One participant described spending “quite a long time […] collating lists” and contacting psychologists, only to find that their waitlists were closed, leading her to feel discouraged: “maybe I don’t need treatment” (P10). Participants considered the Credential and the connect·ed searchable directory as helpful by acting as “an indicator that [clinicians] will be more likely to understand what’s going on for me” (P15). Other participants appreciated the need for ongoing training and supervision to maintain the Credential as they felt this would help keep clinicians up to date with research and treatment approaches. While perceptions of the Credential were positive overall, some participants expressed uncertainty around its ability to guarantee a positive treatment experience. One participant emphasised the importance of clinicians being trauma informed and able to individualise treatment, which she perceived as not necessarily related to whether or not a clinician was credentialed (P9). Even so, these participants still spoke positively about the Credential, with one stating “it’s your best chance of finding someone that knows what they’re talking about” (P13).

While participants generally appreciated that the connect·ed website has a searchable directory of Credentialed Eating Disorder Clinicians, they shared some feedback on its limitations. Some of the participants who lived regionally noted concerns around the availability of credentialed clinicians in their locations, with one noting that even with a “hundred-kilometre radius”, there were no clinicians listed on the connect·ed website or other databases (P12). This meant that participants living regionally may have felt unclear about how to best use the connect·ed website to find clinicians they could access and risked eroding hope. Participants also noted that having up to date details about clinician availability or wait lists would be helpful. Additionally, some participants were interested in additional filters to assist in searching for considerations such as treatment modality offered, treatment philosophy, and lived experience of cultural diversity, living in a larger body, and/or identifying as LGBTQIA+.

## Discussion

The present study’s thematic analysis explored the experiences of ED treatment delivered by credentialed clincians from the perspective of people living with an ED. Overall, the participants perceived the Credential to be important and likely to have a positive impact on treatment outcomes. This perception aligned with their accounts of treatment with credentialed clinicians that were predominantly perceived as helpful. There were two main themes generated from the interviews: treatment experiences contrasting credentialed and non-credentialed clinicians and attitudes towards the Credential. The study found that individuals with lived experience of EDs consistently reported positive experiences with credentialed clinicians, while also feeling positive about the Credential itself. Credentialed clinicians were described as being knowledgeable and having a deeper understanding of EDs, which participants found contributed to a sense of safety in treatment, trust in the therapeutic relationship, and that they mattered. Several participants expressed feeling that the Credential was a sign of a clinician’s commitment to and prioritising of the needs of individuals with EDs, cultivating a sense of hope for their future. Treatment experiences with non-Credentialed clinicians on the other hand, were more variable and included a range of positive and negative experiences.

Participants commonly experienced credentialed clinicians as having the ability to individualise treatment to their needs. This was facilitated by the sense that credentialed clinicians both understood EDs and were able to use a flexible approach to treatment. This preference for knowledgeable, flexible clinicians is aligned with previous research from Gulliksen et al. [24], who found that participants with lived experience of AN preferred clinician qualities such as expertise and taking an active interest in patients over being treated “by the book”. These lived experience treatment preferences for specialist ED treatments to be flexibly tailored to individuals (and families) are consistent with the perspectives of experienced Credentialed Eating Disorder Clinicians [25]. In this study experienced clinicians, perceived supervision CPD as not only supporting clinicians to learn the specifics of ED treatments (“the recipe”) but also to learn how to tailor treatments to individuals (that is, “to cook”) [25].

Some participants reported that their credentialed clinicians worked together as a team, which had positive impacts on their overall treatment by increasing perceived safety and reducing the burden of communication from the person. These reported benefits of clinicians working together as a team are consistent with previous research indicating that collaboration between ED treatment team members is beneficial and preferred by those with EDs [26] and that treatment by a team may lead to improved outcomes compared to treatment by one professional [27].

When reflecting on their attitudes towards the Credential, participants viewed the Credential as a source of hope and validation. The Credential was perceived as a way for the needs of ED treatment seekers to be prioritised by clinicians who choose to become credentialed. Participants described feeling that the Credential indicated a level of commitment from clinicians that helped them to feel safer when seeking treatment. Participants also perceived that the Credential may improve access to treatment by increasing clarity around which clinicians can provide suitable treatment, and through the identification of such clinicians via the connect·ed directory. While participants generally found the connect·ed directory helpful, some participants suggested additional search functions to assist in finding support for those living in regional areas, as well as for finding clinicians with specific interest areas, or personal lived experience that may be important to clients (e.g. experience living in a larger body). This aligns with previous research that found that geographical location can be a barrier to accessing ED treatment [28, 29].

This study provided evidence to demonstrate the impact of the Credential in its early stages, indicating that it may improve treatment experiences for individuals with EDs. The experiences of participants with an ED provided insight into the way that the ongoing training and supervision required by the Credential may improve both therapeutic alliance and safety by preventing negative treatment experiences. To continue to implement and improve the Credential, some considerations were raised. First, as the Credential has been well received by individuals with lived experience of EDs, it would be beneficial to increase awareness of the Credential and connect·ed website as a referral pathway for clients. This may involve encouraging GPs to use the Credential as a referral base, while also encouraging more clinicians to become credentialed. Increased awareness and uptake of the Credential by GPs may also facilitate increased early screening for and identification of ED symptoms in primary care, which has previously been identified as an area for improvement [30]. Second, there is a need for improved access to credentialed clinicians in regional areas. This may involve facilitating the Credential uptake for clinicians living in regional areas, such as by ensuring online training and supervision options are available, and/or by subsidising the credentialing process and ED specific training required to become credentialed for regional clinicians. Third, some participants indicated a need to search for clinicians with specific experience or training, such as an understanding of specific ED comorbidities, working within a Health at Every Size framework, or experience with LGBTQIA + individuals. Further consideration of this preference may help improve access to treatment for populations previously identified as at risk of delayed or missed diagnosis, including those with EDs other than AN or BN [31], and those who are LGBTQIA+, living in larger bodies, or male [30].These considerations could continue to develop the Credential’s capacity to improve treatment access and outcomes for individuals with EDs.

These findings contrast with the general literature investigating consumer views on credentialing. A systematic review in 2012 [32] found very mixed views on accreditation programmes more broadly in health and in particular, a paucity of information on the views and outcomes relevant to people with lived experience. A recent study exploring patient perspectives when selecting an aesthetic surgeon found that a vast majority of patients valued subjective elements such as bedside manner and past patients’ satisfaction, while placing relatively little importance on board certification [33]. The considerable variety in accreditation programs and the diverse nature of health conditions highlights the importance of illness specific research as credentialing may hold different and potentially greater impacts for people with eating disorders in comparison to other areas of healthcare. A key strength of the study was recruitment of participants who had had experienced both a variety of ED diagnoses and treatment from a variety of credentialed clinicians. Participants also experienced a variety of comorbid conditions, including mental health conditions, neurodevelopmental conditions and physical disability. This was important given that there is increased recognition that “comorbidities are the norm” in eating disorders [34]. While the study was primarily qualitative, some descriptive survey data provided additional clarity of information. Participants transcripts were also “member checked” to assess for accuracy. The qualitative nature of the study allowed for greater depth of understanding of the experiences of participants, allowing for a more nuanced understanding of the impacts of the Credential.

Despite the interviewers providing the opportunity for participants to share critical feedback and an inductive approach, so no presumptions were made as to if experiences would be positive or negative, negative themes did not emerge. Given the Credential is in its infancy and the participant experiences of treatment with a credentialed clinician were predominantly positive, it is possible that the sample of participants who volunteered to participate in this study had a tendency towards more positive treatment experiences with a credentialed clinician and that these experiences skewed their recollection of their treatment experiences with clinicians who were not credentialed. We also did not assess participants level of understanding of what was involved in a clinician becoming credentialed and it is possible that the participants awareness of the credentialing process may have influenced their perspective. Further the participant sample reported predominantly experiences of AN, and there was a low representation of individuals from culturally and linguistically diverse backgrounds, and a lack of representation of Aboriginal and Torres Strait Islander peoples. While some participants raised the topic of diversity of gender and sexuality, another limitation is that the study included only female participants. Given that the present study was conducted in the early stages of implementation of the Credential, it is also possible that experiences and perceptions of treatment by credentialed clinicians will change over time. Follow up research is recommended as the Credential becomes more established, to continue to explore and monitor the impact of the Credential on eating disorder treatment experiences and outcomes. Future research may also investigate the impact of the Credential on people from culturally and linguistically diverse backgrounds, as well as on Aboriginal and Torres Strait Islander peoples.

In conclusion, this study found that individuals with lived experience of EDs described predominantly positive experiences with clinicians holding the ANZAED Eating Disorder Credential and feel that the additional training and supervision facilitated their trust in these credentialed clinicians. Participants felt hopeful that the Credential will improve access to treatment and reduce the time spent looking for a suitable clinician, while also safeguarding against negative treatment experiences. However, some participants expressed a desire for clearer pathways to treatment access in regional locations, as well as clear consideration for comorbid conditions and presentations other than AN. While it is evident that individuals with lived experience of EDs value the Credential, it is important that future research endeavours to ascertain the impacts credentialing of clinicians has on actual treatment outcomes in comparison to non-credentialed clinicians.

## Supplementary Information

Additional file 1: Provides the interview guide that was used when interviewing participants with lived experience.

Additional File 2: Exemplar Data Extracts for Themes Identified from Participants’ Semi-Structured Interviews. Additional file 2 provides exemplar data extracts for the themes (Theme 1: A deeper understanding and trust and Theme 2: Hope in treatment) and subthemes (1.1 Trust and safety, 1.2 Seeing the “whole person”, 1.3 Clinicians Teamwork: “My team is solid”, 2.1 A source of hope, 2.2 Potential for improved access to appropriate treatment) identified from semi-structured interviews with participants with lived experience.

## Data Availability

The datasets used and/or analysed during the current study are not publicly available. They may be available from the corresponding author upon reasonable request and in accordance with Human Research Ethics permissions. Permission for the data to be made publicly available is not sought as it would identify participants.

## Abbreviations

ANZAED: Australian & New Zealand Academy for Eating Disorders
ED: Eating Disorder
CPD: Continuing Professional Development
IAEDP: International Association of Eating Disorders Foundation
