# Supplementary material for: A qualitative study of lived experience perspectives and experiences of eating disorder treatment with ANZAED Credentialed Eating Disorder Clinicians
Source: J Eat Disord. 2026 Feb 3;13(Suppl 1):297. doi: 10.1186/s40337-026-01529-6 (PMC12865930; doi:10.1186/s40337-026-01529-6)
Supplement: Supplementary file 1 — Additional file 1: Provides the interview guide that was used when interviewing participants with lived experience. [file 40337_2026_1529_MOESM1_ESM.pdf]

ADDITIONAL FILE 1: Interview Questions

WESTERN SYDNEY UNIVERSITY

Interview Guide: Person Experiencing or Who Has Experienced an Eating Disorder

A selection of questions will be used with each participant and will scaffold between:

- **Experience (e.g. Can you tell me about …?)**
- **Meaning (What does … mean to you?)**

1. Can you tell me your story of your eating disorder?

Prompts:

i. Can you tell me what life was like for you when it all started?

2. Can you please tell us about your experiences in accessing and receiving care for your eating disorder?

Prompts:

i. Can you talk about when you first decided you needed to seek help?
ii. What contributed to your decision to seek care?
iii. When reflecting on your time receiving care, what did you find most helpful in your interactions with clinicians, treatments approaches, etc.
iv. When reflecting on your time receiving care, what did you find the least helpful in your interactions with clinicians, treatment approaches etc.
v. Can you describe any barriers or facilitators you experienced when accessing care?

3. If you used the ConnectED website, can you tell us about that experience?

Prompts:

i. Is your experience what you expected? If not, how did the experience differ from your expectations?
ii. In particular, can you tell me about your experience of using the Find a Treatment Provider search directory?
iii. Explore aspects such as timeliness of accessing care; emotions associated with accessing treatment; availability and opinion on the number of credentialed clinicians within Australia
iv. If you could change anything about the Find a Treatment Provider Directory what would it be?

4. If you accessed treatment from a clinician who is credentialled, can you please tell us about that experience?

Prompts:

i. Was it different from other treatment experiences and in what way?
ii. Do you feel that your cultural beliefs and values were taken into consideration by the Credentialed clinician? If not, please outline what would have been helpful for you.
iii. Tell us about how the Credentialed clinician may have involved other disciplines in your treatment (e.g., dietitian, psychiatrist, paediatrician, social worker)?
iv. What did you most value in your treatment?
v. In what ways did the treatment impact (helped, not helped) you most?

5. If you left (discontinued) treatment at any time, can you tell us about that experience/s?

Prompts:

i. Was it from a credentialled or non-credentialed clinician, or both?
ii. Was it an in-patient, out-patient, day-patient, or other?
iii. What was it about the situation or time for you that prompted you to leave?

6. Based on what you know now, what would you tell a person who is wanting to seek specialised treatment for eating disorders? What might be important for them to know?
