# Supplementary material for: A qualitative study of lived experience perspectives and experiences of eating disorder treatment with ANZAED Credentialed Eating Disorder Clinicians
Source: J Eat Disord. 2026 Feb 3;13(Suppl 1):297. doi: 10.1186/s40337-026-01529-6 (PMC12865930; doi:10.1186/s40337-026-01529-6)
Supplement: Supplementary file 2 — Additional File 2: Exemplar Data Extracts for Themes Identified from Participants’ Semi-Structured Interviews. Additional file 2 provides exemplar data extracts for the themes (Theme 1: A deeper understanding and trust and Theme 2: Hope in treatment) and subthemes (1.1 Trust and safety, 1.2 Seeing the “whole person”, 1.3 Clinicians Teamwork: “My team is solid”, 2.1 A source of hope, 2.2 Potential for improved access to appropriate treatment) identified from semi-structured interviews with participants with lived experience. [file 40337_2026_1529_MOESM2_ESM.pdf]

ADDITIONAL FILE 2: Exemplar Data Extracts for Themes Identified from Participants' Semi-Structured Interviews

Themes	Sub-Themes	Exemplar Extracts
Theme 1: A deeper understanding and trust	Subtheme 1.1 Trust and safety	P10: *"I think it's important to have clinicians who know what they're doing and are kind of keeping up with you know, sort of changes. And and you know the way that the research is is trending and showing things like, I think, rather than just, you know, having studed eating disorders in like the eighties when they did their psychology degree. [...] Yeah, I mean, I think it. It just kind of means like,, this is an area that they like not only find interesting, but are like dedicated to sort of looking about and sort of keeping up with. Yeah, we'll sort of willing to do a little bit extra to to sort of show people that like, I think, yeah, it's it's nice to do. And I think, yeah, it kind of gives that extra level of like trust or you know, sort of see what they're coming from. Yeah."*<br><br>P1: *" I'm really glad that, it kind of makes me feel safer and more confident and knowing that I've got the right person like in my kind of mental health team to help me. And she has been the most helpful in my recovery."*
Theme 1: A deeper understanding and trust	Subtheme 1.2 Seeing the "whole person"	P7: *"Participant: I think you know, one of the main things for me was actually like individualizing the treatment, not just following a, you know, a pathway that's written in a manual and and building trust and connection as well, I think which, you know, is not an easy thing to do"*<br><br>P13: *"I think from my credential clinician, the compassion, the understanding, the openness to not be rigid and to work around like my work, my life, my studies, my trauma stuff. Like all of that."*

Theme 1: A deeper understanding and trust	Subtheme 1.3 Clinicians Teamwork: "My team is solid"	P11: *"And I think it just helped that they all just knew what was going on and I wasn't having to repeat things. And yeah, I think it helped. It probably did help me feel a bit, it it helped me feel quite like, I guess, cared for, because they were very I think proactive in being up to date with my progress and things like that as well. I think the biggest thing was like, when, so my doctor suggested [day programme] to me, and she'd said "yes, she'd discussed it with my dietician and psychologist". I think, knowing it was like everyone's, in everyone's best interest for me. Cause yeah, I, at first I did not want to do it at all. Yeah, I think, having them all be on the same page about it was probably what helped me decide to do it."*<br><br>P6: *"It's important to me that they have personal contact with each other & not only through me. They have regular meetings/phone calls, at least every month to make sure everyone is on the same page and what is taking priority at the moment, e.g. Focusing on nutrition or therapy. [...] How does it feel - it feels safe, and also incredibly frustrating, but in a good way. It feels reassuring to hear the same information coming from all team members. The direction is clear & some of the uncertainty around where to from here/recovery is decreased. I feel held, supported & even though my internal world is in chaos at least my team is solid. "*<br><br>P3: *"Have a unified approach. Okay, so particularly in medical wards, but also in like like public in general, like. You'll have a dietitian, a psych, and a medical doctor, and if you're smart, you can play them off against each other. But also, sometimes nothing happens so like they'll go from one extreme to another"*

Theme 2: Hope in treatment	Subtheme 2.1: A source of hope	P1: *"That sounds amazing. Especially because so many people suffer with eating disorders or even if it's not diagnosable, they suffer with the symptoms. It kind of sounds like getting like a first aid certificate, and having to refresh that, so that you're qualified to at least know the basics around how to treat someone with an eating disorder, that's kind of what it sounds like to me"*<br><br>P9: *"I definitely saw the potential for it, and that's improved a lot. yeah. like any specialization or area of interest is good. The more expertise they can demonstrate in that, the more hope you have that they really know their stuff. So yeah, that's what the benefit is for the credentialed vs non-credentialed."*
Theme 2: Hope in treatment	Subtheme 2.2 Potential for improved access to appropriate treatment	P13: *"But I do send people to the connect.ed website to look for people, because I think it's their best chance. Like it's far from perfect. But it's the best chance. It's like the I-EDPT certification, like there's still idiots that get certified. But it's your best chance of finding someone that knows what they're talking about, and that can fit."*
